# Transposon mutagenesis in oral *streptococcus*

**DOI:** 10.1080/20002297.2022.2104951

**Published:** 2022-07-24

**Authors:** Yixin Zhang, Zhengyi Li, Xin Xu, Xian Peng

**Affiliations:** a State Key Laboratory of Oral Diseases, National Clinical Research Center for Oral Diseases, Chengdu, Sichuan, China; b Department of Cariology and Endodontics, West China School of Stomatology, Sichuan University, Chengdu, Sichuan, China

**Keywords:** Oral streptococci, transposon mutagenesis, next-generation sequencing, bacterial genetics

## Abstract

Oral streptococci are gram-positive facultative anaerobic bacteria that are normal inhabitants of the human oral cavity and play an important role in maintaining oral microecological balance and pathogenesis. Transposon mutagenesis is an effective genetic manipulation strategy for studying the function of genomic features. In order to study cariogenic related genes and crucial biological element genes of oral Streptococcus, transposon mutagenesis was widely used to identify functional genes. With the advent of next-generation sequencing (NGS) technology and the development of transposon random mutation library construction methods, transposon insertion sequencing (TIS) came into being. Benefiting from high-throughput advances in NGS, TIS was able to evaluate the fitness contribution and essentiality of genetic features in the bacterial genome. The application of transposon mutagenesis, including TIS, to oral streptococci provided a massive amount of valuable detailed linkage data between genetic fitness and genetic backgrounds, further clarify the processes of colonization, virulence, and persistence and provides a more reliable basis for investigating relationships with host ecology and disease status. This review focuses on transposon mutagenesis, including TIS, and its applicability in oral streptococci.

## Introduction

Oral streptococci are the microorganisms that colonize the oral surface, comprising the main bacteria in the human oral cavity, where they play important roles in maintaining microecological balance and causing diseases [1,2]. Over 100 oral bacteria have been identified as *Streptococcus* [3]. Currently, oral streptococci have been divided into six groups: *anginosus, bovis, mitis, mutans, salivarius*, and *pyogenic*, based on biochemical testing and 16S rRNA gene sequencing analysis [4,5]. *Streptococcus mutans* is cariogenic and can easily adhere to the surface of teeth, form biofilms, release acidic compounds after carbohydrate metabolism, and enamel demineralization [6]. Conclusive epidemiological evidence has shown that *S. mutans* plays a crucial role in the onset and development of dental caries [7]. The *mitis* and *sanguinis* groups, such as *S. mitis, S. gordonii*, and *S. sanguinis*, are common commensals that can compete with pathogenic bacteria by producing bactericidal hydrogen peroxide for colonisation of the oral cavity [8]. These bacteria are also associated with the formation of biofilms in the oral cavity, which are abundant in both supragingival and subgingival plaques [9,10]. *Streptococcus gordonii* is an initial colonising bacterium on the surface of teeth that can proliferate along with other oral microorganisms, leading to periodontal disease and caries [11]. It can also enter the bloodstream through oral bleeding and increases the risk of invasive infections and systemic diseases, including infective endocarditis [12,13]. The *S. anginosus* group is an important component of the oropharyngeal flora that is commonly associated with various suppurative infections and abscesses in the brain, heart, meninges, liver, spleen, and lung *via* periapical odontogenic lesions and bacteraemia [14]. *Streptococcus constellatus* and *S. intermedius* in dental plaques are associated with the occurrence and development of periodontal disease [15]. In contrast, the *S. salivarius* group, which predominates the oral mucosal surface and saliva, is associated with oral health rather than disease [16].

**Figure 1.**
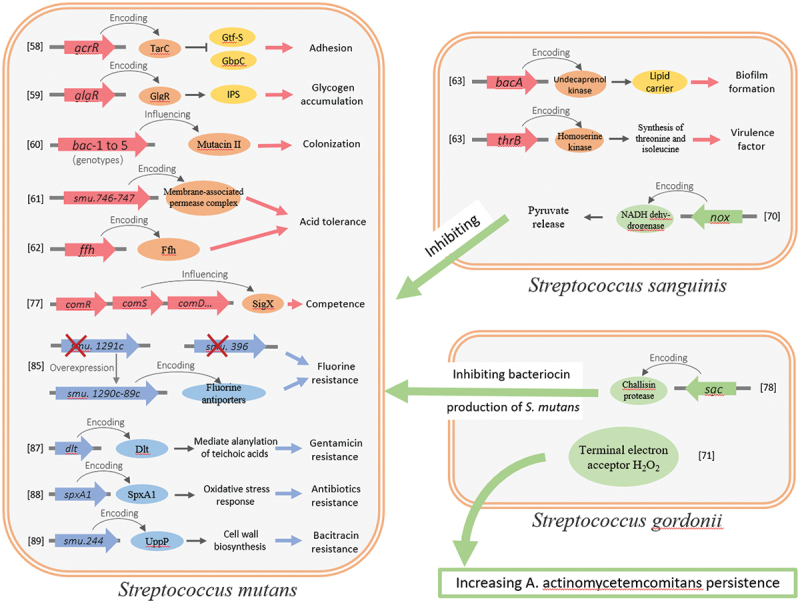
Functional genes identified by transposon mutant library screening in oral *streptococcus*. Red genes are associated with bacterial virulence, green genes with bacteria–bacteria interactions, and blue genes with drug resistance.

Transposon mutagenesis is an effective forward genetic strategy for studying gene function by observing the phenotypic changes in mutated genes. Random mutants in a variety of prokaryotes have been created by using different transposon genes such as Tn*3* derivatives, *IS* (insertion sequence) elements, Tn*7*, Tn*5*, and *mariner*. Since the advent of genome sequencing, techniques such as genetic footprinting, signature-tagged Mutagenesis (STM), transposon site hybridization (TraSH), and scanning Linker mutagenesis (SLM) have been developed [17]. And with the advent of next-generation sequencing (NGS), transposon insertion sequencing (TIS) combines it with large-scale transposon insertion mutations to evaluate the essentiality of genetic features and fitness contribution in the bacterial genome in the saturated random mutant libraries. The four TIS techniques published in 2009 include insertion sequencing (INSeq) in *Bacteroides thetaiotaomicron* [18], high-throughput insertion tracking by deep sequencing (HITS) in *Haemophilus influenzae* [19], transposon sequencing (Tn-Seq) in *S. pneumoniae* [20], and transposon-directed insertion site sequencing (TraDIS) in *S. Typhi* [21]. Those techniques have been widely used in various bacteria to study fitness and virulence, including *Enterococcus faecalis* [22], *Vibrio parahaemolyticus* [23], *Salmonella enteritidis* [24], *Edwardsiella piscicida* [25], *Ralstonia solanacearum* [26] and *Pantoea* [27]. Ultimately, TIS is a key tool for interpreting the rapidly increasing amount of genome sequencing data and is expected to shed light on the function of individual genome features. With the development of transposon technology, TIS has been reviewed from the perspectives of design and analysis [28,29]. Cain et al. discussed recent applications of TIS in answering general biological questions [30]. The present review focuses on oral microorganisms and highlights the application of transposon mutagenesis, including TIS, to oral streptococci, as well as research progress, aiming to better understand the relationship between oral streptococcal phenotype and genotype, which can help clarify the processes of colonization, virulence, and persistence and provides a more reliable basis for investigating relationships with host ecology and disease status. Table 1 and Figure 1 show some articles and conclusions regarding transposon mutagenesis applied to oral streptococci.

**Table 1.** Transposon mutagenesis in oral Streptococci.

Oral *Streptococcus*	Author	Publish Year	Transposon Type	Conclusions	References
*Streptococcus mutans*	Caufield PW, Shah GR, et al	1990	Tn*916*	*Bac-*1to 5 genotypes, responsible for mutacin expression, influences colonization and virulence.	[59]
	Harris GS, Michalek SM, et al	1992	Tn*916*	*glgR* encodes a putative regulator of S. mutans glycogen accumulation.	[58]
	Gutierrez JA, Crowley PJ, et al	1999	Tn*917*	*ffh*, encoding a homologue of the 54 kDa subunit of the signal recognition particle, is involved in resistance to acid stress.	[61]
	Idone V, Brendtro S, et al	2003	Tn*916*	*gcrR*, encoding a putative transcriptional regulator of Gtf-S and glucan binding protein C (GbpC), influences *S. mutans* adherence and subsequent biofilm formation.	[57]
	Król JE, Biswas S, et al	2014	IS*S1*	*smu.746* and *smu.747*, a Putative Membrane Permease Complex, is involved in aciduricity, acidogenesis, and biofilm formation.	[60]
	Jalal N, Tian XL, et al.	2015	*mariner* transposon	*smu.244*, encoding a homologue of UppP, plays important roles in cell wall biosynthesis and bacitracin resistance.	[88]
	Nilsson M, Rybtke M, et al.	2016	*mariner* transposon	*dlt*, mediating alanylation of teichoic acids, is related to gentamicin resistance.	[86]
	Shields RC, O’Brien G, et al.	2017	*mariner* transposon (Tn-Seq)	*comR, comS, comD, comE, cipB, clpX, rcrR, ciaH* and 20 additional genes are identified be required for ComX expression.	[76]
	Shields RC, Zeng L, et al	2018	*mariner* transposon (Tn-Seq)	Essential genes:203 (11%); 295 genes are essential in rich medium; 319 genes are essential in defined medium; >75% genes are potentially required for colonization in the mouse oral cavity.	[49]
	Nilsson M, Jakobsen TH, et al.	2019	*mariner* transposon	*spxA1* is a regulator of genes involved in the oxidative stress response.	[87]
	Yu J, Wang Y, et al.	2020	*mariner* transposon	*smu.396* and *smu.1291c* are related to fluoride resistance phenotype.	[84]
*Streptococcus sanguinis*	Paik S, Senty L, et al.	2005	*mariner* transposon	Genes encoding undecaprenol kinase, homoserine kinase, anaerobic ribonucleotide reductase, adenylosuccinate lyase, and a hypothetical protein are important virulence factors.	[62]
	Redanz S, Treerat P, et al.	2020	*mariner* transposon	*nox*, encoding H2O-forming NADH dehy-drogenase, is essential for oxidative protection and pyruvate release, with other genes such as *dps* and *sodA* having secondary effects.	[69]
*Streptococcus gordonii*	Wang BY, Kuramitsu HK.	2005	Tn*916*	*sgc* inhibits the production of bacteriocin in *S. mutans.*	[77]
	Selleck EM, Gilmore MS.	2016	*mariner* transposon	*S. gordonii* co-infects *A. actinomycetemcomitans* by producing terminal electron acceptor H2O2.	[70]
*Streptococcus pyogenes*	Le Breton Y, Belew AT, et al.	2015	*mariner* transposon (Tn-Seq)	M1T1 5448: Essential genes:227 (12%) M49 NZ131: Essential genes:241 (14%)	[47]
	Chang JC, Federle MJ	2016	*mariner* transposon	*pptAB* encodes the primary transporter for SHP pheromones and other 15 genes are involved in secretion, maturation, detection and degradation of SHP pheromones.	[75]
	Zhu L, Charbonneau ARL, et al.	2017	IS*S1* (TraDIS)	92 genes are required for fitness in saliva.	[64]
	Edgar RJ, van Hensbergen VP, et al.	2019	*mariner* transposon (Tn-Seq)	*gacH* is identified to provide resistance to zinc toxicity and as a putative glycerol phosphate transferase.	[65]
*Streptococcus pneumoniae*	Van Opijnen T, Bodi KL	2009	*mariner* transposon (Tn-Seq)	Essential genes:344 (16%)	[20]
	Verhagen LM, de Jonge MI, et al.	2014	*mariner* transposon (Tn-Seq)	147 genes are potentially required in saliva.	[52]

## Transposon mutagenesis and NGS

Transposons are mobile genetic factors that can move within genomes through ‘cut and paste’ or copy mechanisms. A transposase encoded by a transposon can recognise specific inverted repeat sequences at both ends of the transposon, separate the transposon from adjacent sequences, and insert it into a DNA target site [31]. The most common application of transposons is insertional mutagenesis, which can be used to create libraries of mutant strains. The success of transposon mutant library screening depends on the number of mutants screened and diversity of the library.

Various transposon subsystems have been described [17]. Examples of transposon subsystems include TN*916*, TN*917*, and IS*S1*, which have been used to study oral *Streptococcus* [32–34]. However, certain features of TN*916* and TN*917* prevent the creation of unbiased libraries of randomly inserted transposons. For example, Tn *916* preferentially uses A: T-rich targets but has an insertion hotspot in some bacteria [35]. TN*917* inserts nonrandomly in chromosomes and is far more prevalent in specific DNA regions [36], and IS*S1* mediates transpositions through a replication mechanism, whereby the entire plasmid or sequence of plasmids is integrated into the bacterial genome. Moreover, some bacteria containing endogenous IS*S1* copies can become targets for recombination events [37].

**Figure 2.**
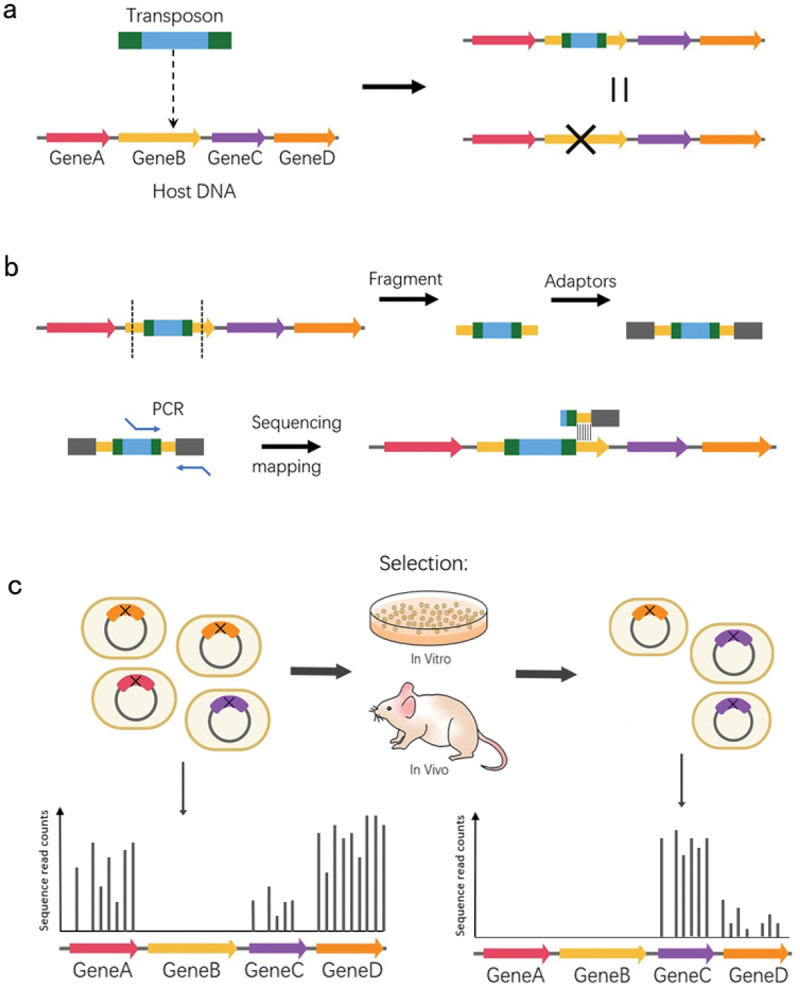
Schema of transposon insertion sequencing. **(a)** Transposon containing inverted repeats at both ends and an antibiotic resistance selection marker is inserted into bacterial genomic DNA to disrupt Gene B. **(b)** Transposon insertion points of each mutant are determined and mapped through breaking, adding adaptors, PCR amplification, and sequencing. **(c)** Bacterial mutant libraries are grown *in vitro* or *in vivo*, and the analysis of the relative abundance of insertion mutants under each growth condition can define the fitness of genetic elements.

Due to the small scale of those transposon subsystems creating mutation libraries, *mariner* or Tn*5* transposon without insertion site bias have been utilized to generate a saturated random mutant library. And TIS combines NGS with large-scale *mariner* or Tn*5* transposon insertion mutations, with which the essentiality of genetic features and fitness contribution in the bacterial genome can be evaluated. Figure 2 shows the basic workflow of the TIS. Briefly, it starts with the construction of a saturated library of random transposon insertions, where the genome of each mutant strain contains a transposon insertion [38,39]. After libraries from various environments are selected, the frequency variation of each inserted mutant is counted by sequencing the overall transposon-flanking region, and these variations are used to estimate the fitness of each mutant. By sequencing before and after selection for a specific condition, changes in the population insertion frequency during selection can define the importance of these genetic elements under that condition. A feature with a reduced insertion frequency is considered important for fitness under these conditions and *vice versa* [40]. Transposon insertion sequencing is a high-throughput approach that reveals phenotypic and genotypic relationships and is applicable to a series of species.

The four major TISs are Tn-Seq, TraDIS, INSeq, and HITS, which vary by transposition, library amplification, sequencing, and bioinformatics methods [41,42]. HITS and TraDIS are applicable to any transposon, but commercial Tn*5* transposon is commonly used. In order to locate the transposon insertion, a series of steps are necessary before sequencing, including shearing DNA, ligating adaptors, amplifying by PCR, affinity purification, and removing extraneous DNA [19,21]. However, the DNA shearing produces a range of fragments sizes, may resulting in PCR bias. INseq and Tn-Seq use the *mariner* transposon exclusively which contains MmeI recognition sites in the terminal inverted repeats. When DNA from mutation library is digested with MmeI, 16 bp of flanking genomic DNA are produced, amplified by PCR after ligating adaptors, and then isolate the 120 bp product by agarose gel or PAGE gel for NGS [18,20]. The *mariner* transposon, which recognises and inserts into TA sites, is applicable to transposition to organisms with low GC in the genome, including oral Streptococci [43]. The *mariner* transposon mutagenesis system can be transposed in streptococci both *in vitro* and *in vivo* [20,44]. In addition, Tn-seq sample preparation protocol is simple, and is easily to isolate the final product of precise length by agarose gel purification, making it an ideal TISs for studying oral *streptococcus.*

The anatomical and physiological characteristics, as well as temperature, humidity, pH, and rich nutrition of the oral cavity, provide a suitable habitat for microorganisms. The resident microorganisms in the oral cavity are numerous and complex. To date, the origin, colonisation, distribution, species, number, succession, and the relationship and dynamic balance between microorganisms and host tissues and cells are not fully understood. Transposon mutagenesis, including TIS, including powerful Tn-Seq, has been widely applied to *Streptococcus* and provided a massive amount of valuable detailed linkage data between genetic fitness and genetic backgrounds. This has significantly contributed to the study of oral *Streptococcus* physiological characteristics, interactions between microbes, and their interrelationships with hosts.

## Uncovering general functions of essential genes and antibacterial drugs development

Transposon mutagenesis combined with NGS has played a significant role in determining the essential genomes of microorganisms [45–47]. Several approaches can be applied to determine the necessary genes using TIS, such as annotation-dependent and independent methods [29]. Essential genes that cannot be mutated determine the basic life processes of bacteria and may be targets of new antimicrobial therapies. Identifying essential genes of pathogenic microorganisms can reveal key genes and pathways to control pathogenic bacteria and the minimum genome of organisms, and these genes might serve as targets for the development of antibacterial drugs.

**Figure 3.**
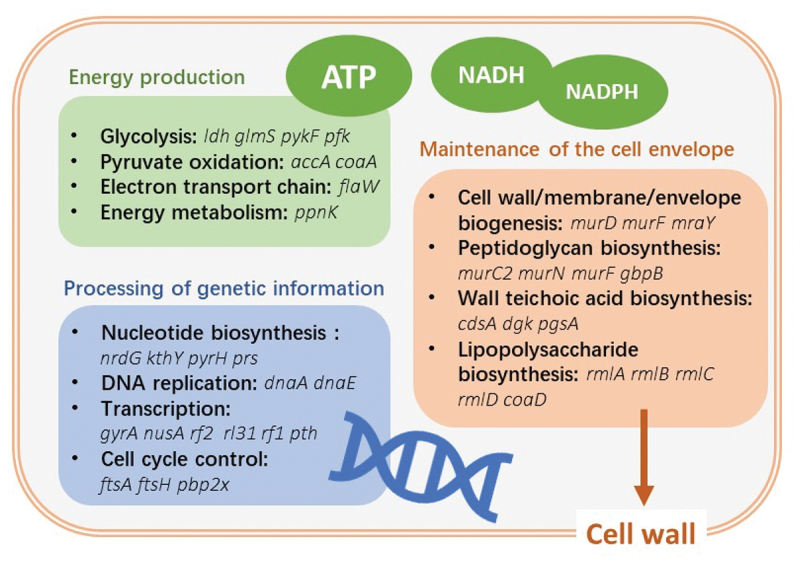
Major biological pathways of essential genes in oral *streptococcus* (based on [48] and [49]).

Van Opijnen et al. first proposed Tn-Seq to determine the fitness of each gene in *S. pneumoniae* and accurately quantified the genetic interactions across the genome [20]. The genomes of *S. sanguinis* and *S. mutans* have also been analysed for gene essentiality. In fact, 9% of the S. *sanguinis* SK36 genome is essential for translation, transcription, glycan biosynthesis, protein folding, sorting, and degradation [48]. In *S. mutans* UA159, 11% of the genome is essential and genes encode products that are closely associated with replication, translation, cell wall synthesis, and lipid metabolism. According to *S. mutans* core genome identified by Cornejo et al, 87% of the essential genes are part of the core genome, and the remaining 13% belong to an accessory genome [49,50]. Predictions indicate that most of the essential genes are part of the core genome; they encode proteins that are needed for basic biological functions and metabolism, and are conserved among strains. Figure 3 shows the major biological pathways of the essential genes in oral streptococci. Some essential genes in the accessory genome, which are also important to the gene-gene network, may be related to coping with unique environmental conditions, such as medium and culture conditions, as well as endogenous metabolic end products. Some genes are condition-specific; that is, they might be necessary for an organism to grow in one environment but not in others. Conditionally essential genes are discrepancies when the mutant libraries are cultured under different conditions, such as in rich or defined medium, acidic conditions or oxidative stress, and rodent models *in vivo* [51,52].

The essential genes conserved between strains and species can be effective targets for antimicrobial agents to control various streptococcal infections. For example, 202 of 218 most essential genes for *S. sanguini* have homologous genes in most other streptococcal genomes [48]. These genes are associated with basic biological processes, including replication, transcription, translation, peptidoglycan synthesis, acetyl coenzyme A biochemical pathways, and lipid synthesis and are highly conserved in most species. Drugs that target specific essential genes found only in one strain, such as those found only in *S. mutans* that encode arginine repressors, superoxide dismutase, l-lactate dehydrogenase, and the shikimate pathway, can control infection without interfering with other beneficial oral bacteria [49,53,54].

## Investigating virulence genes and host adaptation

*Streptococcus* contains a series of virulence factors, including adhesion and surface invasion proteins, as well as proteins for the delivery of toxins to the cell surface and extracellular environment. These factors are related to *Streptococcus* colonisation at different sites, biofilm formation, host tissue destruction, and host immune inflammation [55]. *Streptococcus mutans* is closely related to the occurrence of human dental caries for its physiological properties of adhesive, being acidogenic and aciduric, capable of producing exopolysaccharides [56]. Traditional transposon insertion mutations have been used to explore the genes associated with adhesion [57], glycogen accumulation [58], bacteriocin-like substances synthesis [59], biofilm formation [60], and acid tolerance [61] in *S. mutans* (Table 1).

In niche screening, especially in vivo screening, is extremely valuable for exploring the physiological metabolism of pathogenic microorganisms and discovering the mechanisms of virulence. Streptococcus sanguinis is a common resident of the human oral cavity and one of the major pathogenic bacteria in infective endocarditis. Paik, Sehmi et al. identified six genes associated with virulence 800 mutants in the rat and the rabbit endocarditis model using a modified transposon mutagenesis system (signature-tagged mutagenesis) and dot blot analysis [62]. Next-generation sequencing made it easier and faster to screen virulence factors of bacterial pathogens on a large scale. Through screening the fitness of S. sanguinis mutants in human serum by ORF‐seq (similar to Tn‐seq), 178 mutants with significant abundance changes have been observed. Analysis of the functions of these fitness genes suggests that the virulence factors of S. sanguinis are closely associated with its ability to survive under anaerobic conditions and synthesize cell walls, nucleic acids, and amino acids [63]. In *S. mutans*, fitness determinants required for establishment or persistence under acidic and oxidative stress conditions and in rodent models have also been identified by Tn-Seq. Surprisingly, >75% of the genes in S. mutans UA159 were required for colonization of the mouse oral cavity, possibly because of considerable selection pressure to compete with commensal bacteria for survival during the initial colonization [49,51]. The fitness of group A *streptococcus* (GAS) in human saliva has been studied using TraDIS, and 92 GAS genes were found to be associated with wild-type fitness. Most of the identified genes are related to the transport and metabolism of carbohydrates, inorganic ions, and amino acids [64]. And in *S. pyogenes, gacH* has been identified as being sensitive to phospholipase A2 secreted by the bactericidal enzyme human IIA group and zinc resistant by Tn-seq. The *gacH* gene in the group A carbohydrate (GAC) biosynthesis cluster encodes a new class of glycerophosphoric (GroP) transferases linked to the C6 hydroxyl group of 30% of the GAC N-acetylglucosamine side chain. GroP transferases have also been found in serotype c carbohydrate of *S. mutans*, depending on the presence of their respective *gacH* homologues [65]. This structural change affects the interactions between the host and pathogen, and the development of antimicrobials.

*In vivo* screening is extremely valuable for exploring the physiological metabolism of pathogenic microorganisms and discovering the mechanisms of virulence. However, a major problem with the use of TIS in *vivo* is the effect of bottlenecks. Hampered by the removal or killing of large numbers of bacteria during the establishment of animal models, it is difficult to identify whether these missing mutants are accidental or if their fitness is low [66]. Although this can be partially compensated by optimizing the analytical methods, the main bottleneck effects might irreversibly bias the experiments. Despite its limitations, the application of TIS in animal models has potential value for investigating virulence genes, antimicrobial drugs, and vaccines.

## Understanding bacteria–bacteria interactions

Oral microorganisms do not exist in isolation but constantly interact and form communities with other microorganisms, and these interactions are considered important factors in the formation of disease states. Interactions among colonised oral microbes can continuously accelerate or inhibit biofilm development, and transposon mutagenesis offers the potential to identify such interactions.

*Streptococcus sanguinis* and *S. gordonii* generate H_2_O_2,_ which inhibits the growth of *S. mutans* through pyruvate oxidase encoded by *spxB* [67,68]. The catabolite control protein A (CcpA) represses *spxB* expression and H_2_O_2_ release [68]. While the *ccpA* deletion mutants of *S. gordonii* and *S. sanguinis* could directly detoxify H_2_O_2_ *via* pyruvate release and confer protection in trans to other bacteria. Targeted and transposon mutagenesis suggests that *nox*, which is presumed to encode H_2_O-forming NADH dehydrogenase, is essential for oxidative protection and pyruvate release, with other genes such as *dps* and *sodA* having secondary effects [69]. This study revealed a novel aspect of the competitive interaction between pathogens and oral commensals and offers a direction for further study of the mechanisms underlying the varying degrees of inhibition potential between strains of commensal oral streptococci that produce H_2_O_2_. A new mechanism that leads to the combined growth of oral microbes *Aggregatibacter actinomycetemcomitans* and *S. gordonii* has also been revealed by Tn-Seq. The latter co-infects *A. actinomycetemcomitans* by producing the terminal electron acceptor H_2_O_2_, which changes the growth mode of *A. actinomycetemcomitans* from anaerobic to aerobic, increasing its persistence. This interaction is referred to as ‘cross respiration’, implying that influencing the *S. gordonii* antibacterial regimen helps combat such co-infections [70].

Quorum sensing is a communication method to coordinate a response in a population employed by bacteria, and the study of genetic competence is a model pathway to explore intercellular communication, especially in Streptococcus. Genetic competence is required for obtaining extracellular DNA and also has a significant impact on the expression of virulence-related features, biofilm formation, and stress tolerance [71]. Extensive research has identified two competence-activating signaling systems, the XIP/ComRS system and CSP/ComCDE the system [72]. *Streptococcus mutans*, which containing both systems, has become an attractive model to study the two signaling systems. In S. mutans, transposon insertion mutated strains (i.e. *comR, oppABCDF, comX*, and *irvR*) have been defined to have great fitness in the mouse oral cavity by Tn-Seq [49]. ComR and the OppABCDF are required for the activation of transcription of *comX* (*sigX*), which encodes the alternative sigma factor that controls late competence gene activation, and IrvR is an important regulator for genetic competence. Previous studies have shown that the virulence of *S. pneumoniae* has been attenuated in a Δ*comX* mutant, for loss of induction of the allolytic genes *cibAB* and *cbpD* [73]. But Orthologs of *cibAB* and *cbpD* are not present in the S. mutans. And the production of ComX has been demonstrated to lead to growth arrest and cell lysis of S. mutans [74], which may account for the fitness enhancements of these mutants. In S. pyogenes, transposon mutagenesis screening identified the ABC transporter PptAB, which plays an important role in short hydrophobic peptide (SHP) pheromone output through the Rgg2/Rgg3 pathway. However, in S. mutans, removal of pptAB only partially disrupted XIP signaling suggesting PptAB is not key to the ComRS signaling pathway and the secretion of XIP may have a secondary secretion pathway [75]. Shields, Robert C et al created a transposon insertion library containing the *comX* promoter in S. mutans, and novel genes associated with competence development have been identified by Tn-seq, and 20 genes have been identified and characterised in addition to known genes associated with ComX expression. These data also highlight *DivIB* may be the focus of future studies on the crosstalk between ComRS and ComCDE systems in S. mutans [76]. On the other hand, Tn916 mutagenesis has shown that the *sgc* gene of *S. gordonii* inhibits the production of bacteriocin regulated by ComCDE system in *S. mutans* [77].

Mutant strains in a mutant pool may interact to be complemented, thereby concealing their virulence defects and changing their fitness. Droplet Tn‐Seq has been developed to achieve independent growth by using microfluidic technologies to encapsulate each transposon mutant into a growth medium-in-oil droplet [78]. Through defining single-cell fitness in a genome-wide by dTn-seq, it is possible to further explore interbacterial interactions and bacterial microcolony formation. And combining TIS with other high-throughput technologies, such as RNA-Seq and metabolomics, can reveal competition for environmental resources among microorganisms and important new pathways for microbial community interactions. The combination of Tn-Seq with RNA-Seq has been applied to identify genes that are important for the growth of *E. faecium* in human serum [79] and explore the interaction between *Escherichia coli* and microorganisms in cheese environments [80]. The microbial community structure and function depend on complex interactions that are both competitive and beneficial. The increasing complexity of the community leads to changes in the genetic requirements for microbial interactions. The online application ShinyOmics has been developed to allow rapid collaboration in the analysis and exploration of the massive accumulation of bio-omics data [81]. Similar methods and analytical tools should be used to explore the complex and rich interactions among oral microorganisms.

## Identifying genes involved in drug resistance

Transposon mutation can be utilized to study the drug sensitivity of different mutants, which helps better understand the development of bacterial resistance. Fluoride exerts significant anticaries effects by inhibiting demineralisation, enhancing remineralisation, and inhibiting bacterial growth, which play important roles in oral health [82]. *Streptococcus mutans* is the major pathogen causing dental caries, and the widespread use of fluoride might lead to the emergence of bacteria that are resistant to fluoride [83]. Transposon insertion mutants of *S. mutans* were constructed and a library was screened to identify and characterise genes associated with fluorine tolerance. The results showed that *smu.1289c-90c* overexpression combined with *smu.396* deletion resulted in higher fluorine resistance in the *smu.1290c-89c* operon encoding fluorine antiporters [84,85]. Screening important genes of the *S. mutans* transposon mutant library for biofilm-related antibiotic resistance indicated that the *dlt* gene is associated with gentamicin resistance in *S. mutans* biofilms. The expression of *dlt* genes mediates the alanylation of teichoic acids, and the negative charge on the surface of *dltA* mutants is greater than that of the wild-type, which leads to reduced tolerance to positively charged gentamicin [86].

Drug-bacterial interactions are not only limited to a drug and its direct target but also to drug-induced pressure that seems to resonate through bacteria, resulting in selective pressure. Transposon mutagenesis cannot directly assay drug targets such as DNA replication, cell wall synthesis, or protein synthesis. However, detecting the relative changes in the number of genes inserted into transposons during drug exposure helps to reveal this complex multifactorial process. Transposon mutagenesis has shown that the antimicrobial tolerance of *S. mutans* biofilms also depends on an oxidative stress response mediated by the SpxA1 protein, which functions as a transcription factor [87]. Screening a transposon mutant library revealed that *SMU.244* encodes a homologue of undecaprenyl pyrophosphate phosphatase, which plays important roles in bacitracin resistance and cell wall biosynthesis in *S. mutans* [88].

Sensitivity profiles constructed using Tn-Seq have shown that the two strains of *S. pneumoniae* use several genes to resist stress triggered by daptomycin, including genes important for membrane integrity, protein conversion, and potassium uptake. The activity patterns of antibiotics have been partially uncovered by confirming numerous genotype-phenotype relationships, investigating temporal gene expression, and mapping genetic interactions [89]. Viridans group streptococci are important normal bacteria in humans; they are most abundant in the oral cavity and are considered the causative pathogens of infective endocarditis, septicaemia, meningitis, and other serious infections. We predict that TIS will be used to reveal oral streptococcal genes and networks related to drug resistance and develop new therapies for targeting drug-resistant bacteria.

## Next frontiers

Transposon mutagenesis and NGS have become the preferred methods for large-scale detection of genotype-phenotype interactions because of their high-throughput capability and sensitivity to small differences in fitness. The functions of most nonessential genetic components in organisms can be explored using this technique under various environmental conditions. TIS also works in conjunction with other modern technologies such as RNA-Seq. Since its development, TIS has been applied to studies of *in vitro* and *in vivo* models to explore the fitness of genes, shed new light on studied biological processes, and begin to understand how genotypes influence pathogenicity at the genome level.

Although transposon sequencing has some applicability, it still has some limitations. Traditional TIS is mainly used to study the functions of nonessential genes and identify essential genes. However, libraries with large numbers of mutants have a bottleneck effect; insertion mutants may be randomly lost during selective growth for reasons unrelated to fitness, especially in animal models. Liu et al. designed single-guide RNA (sgRNA) sequences targeting core genes identified by Tn-Seq and developed an IPTG-induced CRISPR interference (CRISPRi) system for functional studies of essential *S. pneumoniae* D39V genes *in vitro* [90]. Bosch et al. developed a CRISPRi platform for *S. thermophilus* to provide a genome-level assessment of gene vulnerability, which links the degree of gene inhibition to its effect on fitness [91]. Liu et al. recently developed a doxycycline-induced CRISPRi system and constructed a pooled CRISPRi library that targets almost all operons of *S. pneumoniae* D39V and can be easily combined with Illumina sequencing (CRISPRi-Seq) [92]. By selecting a sgRNA for each operon, CRISPRi-Seq was used to assess bottlenecks and identify pneumococcal genes that are important in a murine pneumonia model. Genome-wide CRISPR screening can be used to systematically investigate gene functions. However, an sgRNA library is large, and its synthesis is expensive. Jiang et al. used the CRISPR-CAS adaptation mechanism of *S. pyogenes* to develop CRISPR adaptation-mediated library manufacturing, which transforms bacterial cells into ‘factories’ that generate hundreds of thousands of CRISPR RNAs, covering 95% of all targeted genomic sites [93]. However, this method also produces numerous mutants resulting in bottlenecks. Moreover, when operons contain multiple essential genes, the CRISPRi system results in polarity effects that inhibit the expression of downstream genes. The insertion of transposons within operons in Tn-Seq, owing to the lack of transcriptional terminators, allows for read-through transcription and, thus, minimises polarity effects. With the development of TIS, the functions of essential genes can be studied using transposon libraries with outwards promoters that promote gene overexpression [94]. Along with technical and analytical development, the bottleneck of saturated libraries between different conditions gradually decreases. In general, TIS is simple to operate and inexpensive and is still a powerful tool for high-throughput quantitative studies of microbial genotypes influencing their phenotypes. The CRISPRi system is indispensable for the functional study of essential genes in microorganisms.

## Conclusions and perspectives

Transposon mutagenesis could lead to a better understanding of microbial interactions by providing a better annotation for more types of oral *Streptococcus* phenotypes. Studies on gene function should be further advanced using other basic experimental methods, such as biochemical studies and microscopy, to understand the specific mechanisms of gene function. Furthermore, TIS can also be combined with other methods, such as RNA sequencing, microfluidics, and CRISPRi, to explore microbial interactions *in vitro* and *in vivo* and discover new and important toxicity properties that will deepen our understanding of oral health homeostasis and disease dysregulation. As TIS technology has become more advanced and other types of data are combined for analysis, data analysis tools and visualisation have become increasingly important. We predict that transposon mutations and NGS technologies will continue to be developed for applications to address diverse and complex biological questions.
